# Nicotinamide mononucleotide supplementation rescues mitochondrial and energy metabolism functions and ameliorates inflammatory states in the ovaries of aging mice

**DOI:** 10.1002/mco2.727

**Published:** 2024-09-30

**Authors:** Jinghui Liang, Feiling Huang, Xueyu Hao, Peng Zhang, Rong Chen

**Affiliations:** ^1^ Department of Obstetrics and Gynecology, Peking Union Medical College Hospital, Chinese Academy of Medical Sciences & Peking Union Medical College National Clinical Research Center for Obstetric & Gynecologic Diseases Beijing China; ^2^ Beijing Key Laboratory for Genetics of Birth Defects, Beijing Pediatric Research Institute, Beijing Children's Hospital, Capital Medical University National Center for Children's Health Beijing China; ^3^ MOE Key Laboratory of Major Diseases in Children, Beijing Children's Hospital, Capital Medical University National Center for Children's Health Beijing China; ^4^ Beijing Key Laboratory for Genetics of Birth Defects, Beijing Pediatric Research Institute; MOE Key Laboratory of Major Diseases in Children; Rare Disease Center, Beijing Children's Hospital, Capital Medical University National Center for Children's Health Beijing China

**Keywords:** infertility, nicotinamide adenine dinucleotide (NAD^+^), nicotinamide mononucleotide, ovarian aging

## Abstract

Noninvasive pharmacological strategies like nicotinamide mononucleotide (NMN) supplementation can effectively address age‐related ovarian infertility by maintaining or enhancing oocyte quality and quantity. This study revealed that ovarian nicotinamide adenine dinucleotide levels decline with age, but NMN administration significantly restores these levels, preventing ovarian atrophy and enhancing the quality and quantity of ovulated oocytes. Improvements in serum hormone secretion and antioxidant factors, along with decreased expression of proinflammatory factors, were observed. Additionally, a significant increase in the number of ovarian follicles in aging individuals was noted. Scanning electron microscopy data indicated that NMN significantly alters the density and morphology of lipid droplets and mitochondria in granulosa cells, suggesting potential targets and mechanisms. Transcriptomic analysis and validation experiments collectively suggested that the beneficial effects of NMN on aging ovaries are mediated through enhanced mitochondrial function, improved energy metabolism, and reduced inflammation levels. Our results suggest that NMN supplementation could improve the health status of aging ovaries and enhance ovarian reserve, offering new insights into addressing fertility challenges in older women through assisted reproductive technology.

## Graphical abstract

Supplementing with NMN in vivo reverses ovarian aging by: (1) increasing ovarian NAD+ levels and serum antiaging effects; (2) inhibiting ovarian atrophy, enhancing hormone secretion, modulating mitochondrial and lipid droplet properties, and improving ovarian health; (3) increasing the number of oocytes at various developmental stages, improving the quality and quantity of ovulatory oocytes; (4) enhancing mitochondrial function and energy metabolism while reducing ovarian inflammation.

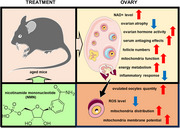

## 1 INTRODUCTION

Female fertility refers to the ability of women to produce oocytes, undergo fertilization, and carry a fetus. Typically, female fertility reaches its peak at the age of 24 years, declines after the age of 30 years, and accelerates after the age of 37 years. Most women experience a natural termination of their reproductive capacity approximately 10 years before menopause, with complete depletion of oocyte reserves in the ovaries around the age of 50 years.^1^, ^2^ The primary cause of decreased fertility in older women is ovarian aging.^3^ During ovarian aging, the number and quality of follicles decrease,^4^ ovarian secretory function decreases, and the follicle pool is depleted of unstimulated follicles.^5^ Although assisted reproductive technology (ART) can assist infertile couples in achieving pregnancy, advanced maternal age poses challenges due to the decline in the number and quality of ovarian follicles, resulting in a reduced number of high‐quality oocytes. Consequently, even with the aid of ART, obtaining desirable outcomes becomes more difficult.^6^, ^7^ Despite the significant clinical consequences of ovarian aging, there are currently no treatments that address the decline in age‐related ovarian reserve, and understanding of the underlying molecular mechanisms is insufficient,^8^ hindering progress in the treatment of ovarian aging. The key to improving fertility in older women is to study the mechanisms of ovarian aging and, based on those studies, to develop methods for delaying the process of ovarian aging and improving the number and quality of follicles in the ovaries.^9^, ^10^

The ovaries are complex organs containing numerous cells that play different functional roles and have significant heterogeneity, and their aging is influenced by various factors.^11^, ^12^, ^13^ Mitochondrial function and ovarian aging are closely related. Mutations in mitochondrial DNA, impaired oxidative phosphorylation, increased oxidative stress, alterations in mitochondrial quality control, inefficient metabolic and clearance processes in mitochondria, and dysregulation of mitochondrial dynamics are potentially associated with ovarian aging.^14^, ^15^, ^16^, ^17^, ^18^ In the ovary, lipids are stored predominantly as triglycerides within lipid droplets. Upon the conversion of triglycerides to free fatty acids, they can be transported into mitochondria through the action of carnitine palmitoyltransferase types 1 and 2 (CPT1 and CPT2) and their cofactors. Through the fatty acid β‐oxidation pathway, ATP is subsequently produced, thereby supplying the energy necessary for oocyte maturation and embryonic development.^19^, ^20^, ^21^ A number of processes related to reproduction are affected by inflammation, including oocyte maturation, ovulation, embryo implantation, and parturition.^22^, ^23^, ^24^ The body's tissues and organs experience sluggish, low‐grade, progressive inflammation as we age.^25^ More recently, research has indicated that inflammation is a critical marker of ovarian stromal aging.^26^ According to research conducted using animal models, female fertility is enhanced when tumor necrosis factor (TNF) receptors are absent, and mice deficient in interleukin‐1α (IL‐1α) exhibit extended ovarian lifespan.^27^, ^28^ Therefore, improving mitochondrial function and reducing inflammation in aging ovarian cells may be possible approaches to delaying age‐related decreases in fertility.^7^

Nicotinamide adenine dinucleotide (NAD^+^) levels in the body decline with increasing age, contributing to the progression of age‐related diseases.^29^ Nicotinamide mononucleotide (NMN) is an effective precursor of NAD^+^ in the body. Inside cells, NMN reacts with ATP to generate NAD^+^, which mediates cellular biological processes such as energy metabolism, DNA damage repair, epigenetic changes, regulation of gene expression, and inflammation. Therefore, restoring NAD^+^ levels in the body may be an effective intervention for combating ovarian aging. Research has demonstrated that NMN treatment can improve muscle insulin sensitivity in prediabetic women^30^ and enhance aerobic capacity in recreational runners.^31^ NMN has also been shown to improve oocyte quality and to increase fertility and postpone ovarian aging in animal models.^32^, ^33^, ^34^ While the application of NMN in the context of aging has advanced significantly, its effects on female ovarian aging and the processes underlying it remain unclear.

In this work, we found that supplementation with NMN in vivo can restore NAD^+^ levels in the ovaries of aged mice, inhibit ovarian atrophy, and improve ovarian hormone secretion, thereby strengthening the antiaging effects of serum in older mice. NAD^+^ supplementation also contributes to enhancing the quality of ovulated oocytes and increasing the number of ovarian follicles at various stages of development. The ultrastructures of lipid droplets and mitochondria in ovarian granulosa cells improved after NMN treatment, providing a structural basis for the observed reversal in ovarian aging. Through transcriptomic analysis and subsequent validation experiments, we further determined that NMN treatment enhances mitochondrial function and cellular energy metabolism. It also regulates inflammatory and immune‐related pathways as well as intercellular interactions, reducing ovarian inflammation levels. This ultimately reduces the effects of ovarian aging and enhances ovarian health status.

## 2 RESULTS

### 2.1 Short‐term NMN supplementation enhances ovarian NAD^+^ levels and improves the quality of ovulation‐induced oocytes in aged mice

**FIGURE 1:**
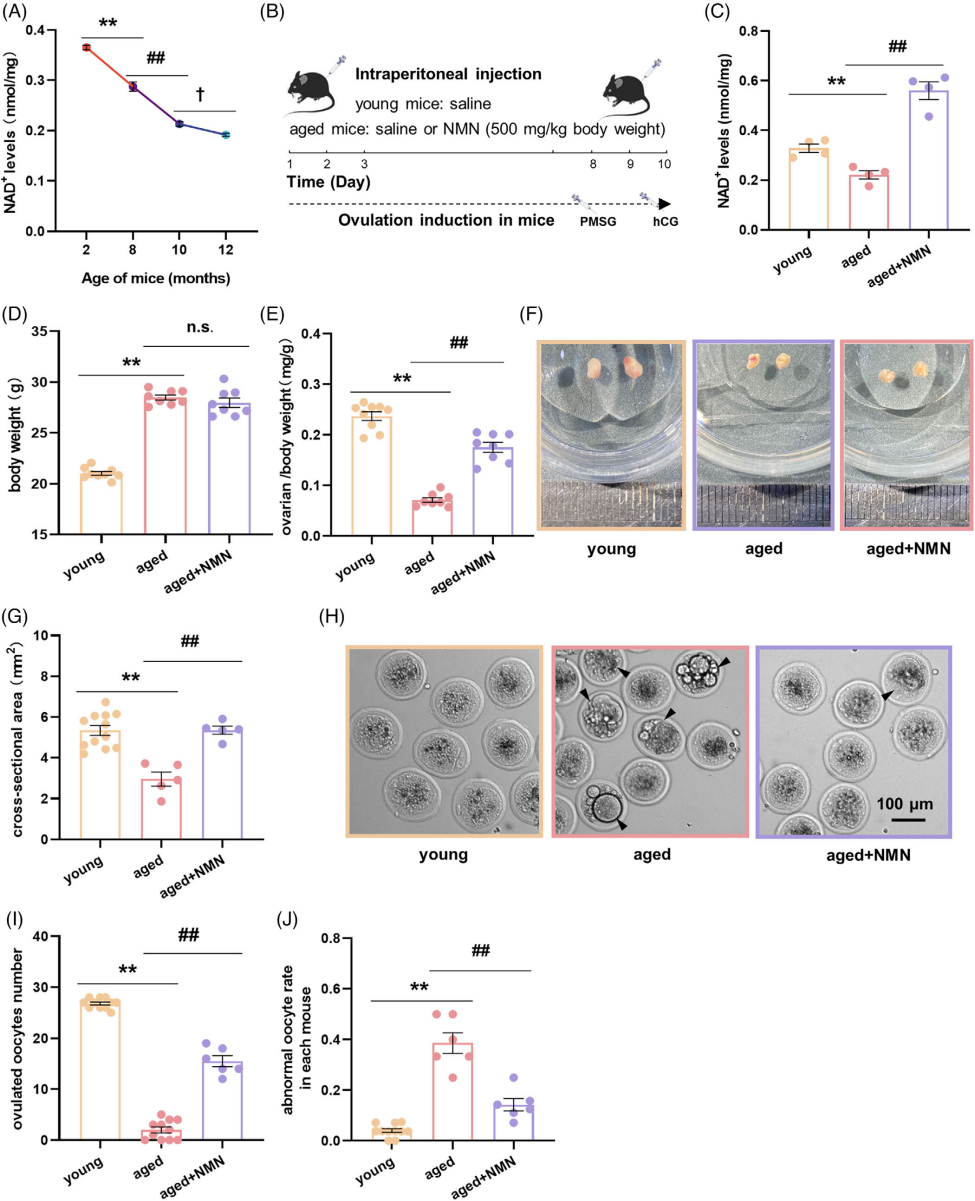
Short‐term supplementation with nicotinamide mononucleotide (NMN) significantly increases ovarian nicotinamide adenine dinucleotide (NAD+) levels, inhibits ovarian atrophy, and improves the quality of ovulation‐induced oocytes in aged mice. (A) NAD^+^ levels in the ovaries of mice at different ages (*n* = 3 mice at 2, 8, and 12 months, 6 mice at 10 months). (B) Timeline for short‐term intraperitoneal injection of NMN and ovulation‐inducing hormones in mice. (C) NAD^+^ levels in the ovaries of animals in the young, aged, and aged + NMN groups (*n* = 4 mice per group). (D) Body weight of the animals in the young, aged, and aged + NMN groups (*n* = 9 mice in the young group, 8 mice in the aged and aged + NMN groups). (E) Ovarian weight/body weight ratio in the mice in the young, aged, and aged + NMN groups (*n* = 8 mice per group). (F) Representative images of ovaries from the animals in the young, aged, and aged + NMN groups. (G) Statistical analysis of ovarian cross‐sectional area in the young, aged, and aged + NMN groups (*n* = 12 mice in the young group, 5 mice in the aged and aged + NMN groups). (H) Representative images of ovulation‐induced oocytes obtained from the mice in the young, aged, and aged + NMN groups. Black arrows indicate abnormal oocytes. Scale bar, 200 µm. (I) Quantification of the number of oocytes obtained from the ovaries of young, aged, and aged + NMN mice (*n* = 11 mice in the young and aged groups, 6 mice in the aged + NMN groups). (J) Percentage of abnormal oocytes obtained from young, aged, and aged + NMN mice (*n* = 11 mice in the young group, 6 mice in the aged and aged + NMN groups). The data are presented as the mean ± SEM. *, #, or †, *p* < 0.05; ** or ##, *p* < 0.01.

First, we aimed to determine the relationship between age and ovarian NAD^+^ levels and whether supplementation with NMN, a precursor of NAD^+^, could increase ovarian NAD^+^ levels and improve the health status of aging ovaries. We measured the NAD^+^ content (Figure 1A), body weight (Figure S1A), and ovarian/body weight ratio (Figure S1B) of mice 2, 8, 10, and 12 months of age. We found that ovarian NAD^+^ levels declined progressively with age, with a more rapid decline before 10 months and a relatively slower decline between 10 and 12 months. To evaluate the impact on NMN, we designed a short‐term NMN administration regimen in which mice 2 months of age and mice 12 months of age were used, as depicted in Figure 1B. The young group received intraperitoneal injections of saline, and the aged group received injections of saline or NMN saline solution in which each injection contained 500 mg of NMN per kg of body weight. NMN supplementation effectively countered the decrease in ovarian NAD^+^ concentration in aged mice (Figure 1C). Although short‐term NMN supplementation did not significantly affect body weight in the aged group (Figure 1D), it significantly restored the ovarian/body weight ratio in aged mice (Figure 1E). Analysis of two‐dimensional images of intact mouse ovaries (Figure 1F) revealed that NMN supplementation significantly increased the cross‐sectional area of ovaries in aged mice (Figure 1G).

Using this model, we studied mouse ovulation‐induced oocytes (Figure 1H). We observed that NMN treatment notably increased their number in the aged + NMN group relative to the number in the aged group (Figure 1I). NMN treatment also reduced the number of morphological defects (such as abnormal morphology, unevenly distributed cytoplasm, multinucleation, and cytoplasmic vesicle abnormalities) observed in the oocytes (Figure 1J). Additionally, we studied the quality of the ovulation‐induced oocytes and found that supplementation with NMN improved the distribution of mitochondria in the ovulation‐induced oocytes of aged mice (Figure S2A,B) and increased the mitochondrial membrane potential (Figure S2C,D), and reduced the level of cellular reactive oxygen species (ROS) (Figure S2E,F) in these oocytes.

### 2.2 Long‐term administration of NMN enhances antiaging properties in aged mice

**FIGURE 2:**
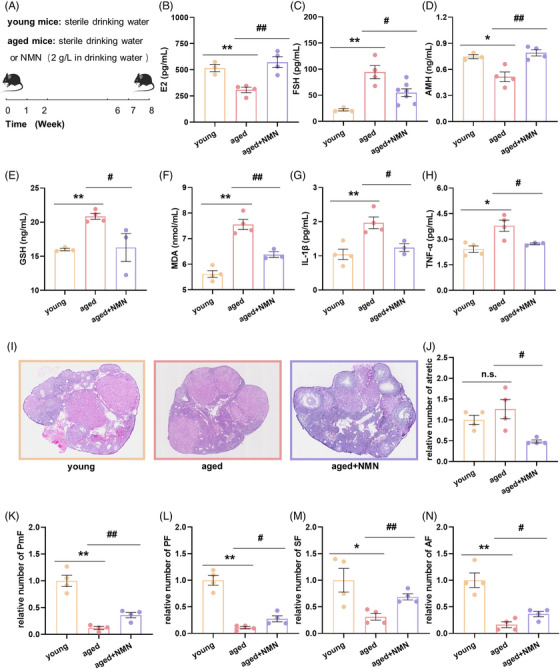
Long‐term NMN treatment enhances serum antiaging effects and increases follicle numbers in aged mice. (A) Timeline of long‐term administration of NMN to mice via their drinking water. (B) Serum estrogen (E2) levels in the mice in the young, aged, and aged + NMN groups (*n* = 3 mice in the young group, 4 mice in the aged and aged + NMN groups). (C) Serum follicle‐stimulating hormone (FSH) levels in the mice in the young, aged, and aged + NMN groups (*n* = 3 mice in the young group, 4 mice in the aged group, and 7 mice in the aged + NMN group). (D) Serum anti‐Müllerian hormone (AMH) levels in the mice in the young, aged, and aged + NMN groups (*n* = 3 mice in the young group, 4 mice in the aged and aged + NMN groups). (E) Levels of the serum antioxidant factor glutathione (GSH) in the mice in the young, aged, and aged + NMN groups (*n* = 3 mice in the young group, 4 mice in the aged group, and 3 mice in the aged + NMN group). (F) Levels of the serum antioxidant factor malondialdehyde (MDA) in the mice in the young, aged, and aged + NMN groups (*n* = 4 mice in the young group, 4 mice in the aged group, and 3 mice in the aged + NMN group). (G and H) Levels of the serum proinflammatory factors interleukin‐1 beta (IL‐1β) and tumor necrosis factor‐alpha (TNF‐α) in the mice in the young, aged, and aged + NMN groups (*n* = 4 mice in the young group, *n* = 4 mice in the aged group, and *n* = 3 mice in the aged + NMN group). (I) Representative images of hematoxylin and eosin (HE) stained ovarian sections from the mice in the young, aged, and aged + NMN groups. Scale bar, 500 µm. (J–M) Follicle dynamics in each group of mice (*n* = 4 mice per group). PmF, primordial follicle; PF, primary follicle; SF, secondary follicle; AF, antral follicle. The data are presented as the means ± SEMs. * or #, *p* < 0.05; ** or ##, *p* < 0.01.

We designed a long‐term administration method in which NMN was administered to aged mice for 8 consecutive weeks via their drinking water (Figure 2A). We used this method to monitor the impact of prolonged treatment with NMN on the ovaries and the general health of aged mice. We chose to use serum hormones, antioxidants, and inflammatory factors as our assessment markers, given their extensive application and frequent citation in research. We selected these markers in an attempt to precisely measure the potential impacts of NMN administration on the health status of mice. NMN treatment significantly elevated estrogen (E2) levels, decreased follicle‐stimulating hormone (FSH) levels, and increased anti‐Müllerian hormone (AMH) levels (Figure 2D) in the serum of aged mice (Figure 2B–D), suggesting that NMN treatment improved ovarian hormone secretion. Measurement of the levels of the antioxidant factors glutathione (GSH) (Figure 2E) and malondialdehyde (MDA) (Figure 2F) in the animals’ serum revealed that the levels of these factors decreased significantly after NMN treatment, indicating that NMN treatment reduced oxidative stress and promoted a more favorable health status. Analysis of the serum concentrations of the proinflammatory factors IL‐1β (Figure 2G) and TNF‐α (Figure 2H) revealed that NMN treatment suppressed the increased inflammation induced by aging, as indicated by the decreased concentrations of these proinflammatory substances in the serum. The results indicate that NMN treatment improved the overall health status of the mice and enhanced ovarian hormone secretion.

Next, we used hematoxylin and eosin (HE) staining to study the impact of NMN therapy on the follicle numbers in aged mice. Frozen sections of ovaries from the animals in the young, aged, and aged + NMN groups were stained with HE at a thickness of 8 µm per section, and the number of follicles at each developmental stage was counted in every 10th section (Figure 2I). We discovered that the ratio of atretic follicles in the aged + NMN group was much lower than the ratio of atretic follicles in the aged group (Figure 2J), indicating that NMN caused many follicles in aging ovaries to mature rather than become atretic. The proportion of developing follicles at each developmental stage (PmF, primordial follicle; PF, primary follicle; SF, secondary follicle; and AF, antral follicle) was consistently greater in the young group than in the aged group (Figure 2K–N), indicating continuous depletion of the follicle pool with age. In the ovaries of aged mice, NMN treatment significantly increased the relative ratio of follicles at all developmental stages (Figure 2K–N) and inhibited follicular atresia (Figure 2J). Therefore, long‐term supplementation with NMN significantly improved the ovarian reserve in aged mice.

### 2.3 NMN improves lipid droplet content and mitochondrial ultrastructure in ovarian granulosa cells

**FIGURE 3:**
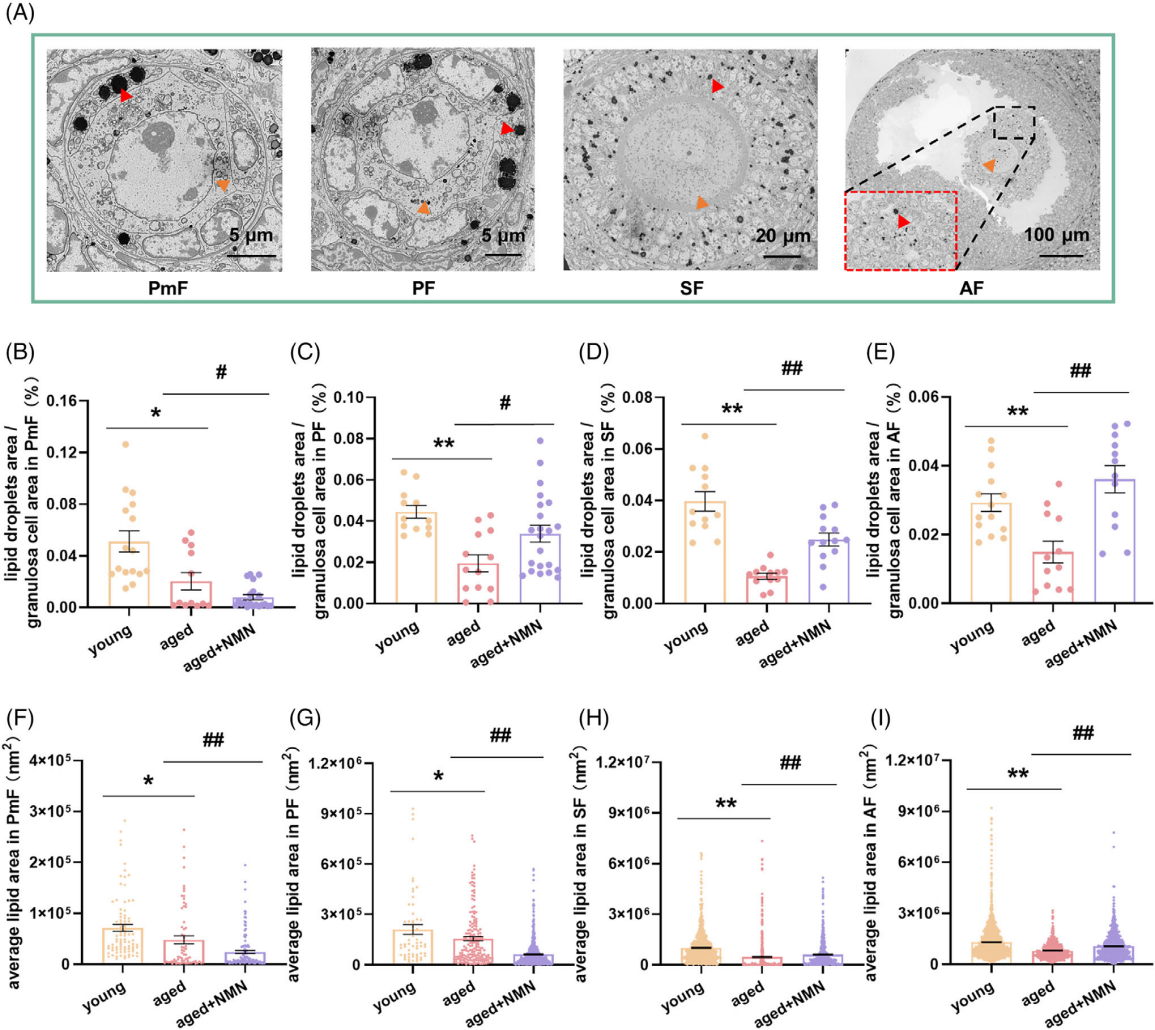
Effect of NMN supplementation on lipid droplets in mouse granulosa cells. (A) Representative scanning electron microscope images of PmF, PF, SF, and AF in mouse ovaries. Red arrows indicate lipid droplets present in the granulosa cells; orange arrows indicate oocytes. The red dashed box in the AF image shows an enlarged view of the area within the black dashed box. The scale bars are 5, 5, 20, and 100 µm. (B–E) Quantification of the lipid droplet content of PmF, PF, SF, and AF granulosa cells in the ovaries of mice in the young, aged, and aged + NMN groups (PmF: *n* = 16 follicles in the young group, 12 follicles in the aged group, and 20 follicles in the aged + NMN group; PF: *n* = 12 follicles in the young group, 13 follicles in the aged group, and 22 follicles in the aged + NMN group; SF: *n* = 12 follicles in the young and aged groups, 13 follicles in the aged + NMN group; AF: *n* = 14 follicles in the young group, 12 follicles in the aged and aged + NMN groups. Four mice in each group were sampled. (F–I) Quantification of the average area of individual lipid droplets in PmF, PF, SF, and AF granulosa cells of mice in the young, aged, and aged + NMN groups (PmF: *n* = 85 lipid droplets in the young group, 68 lipid droplets in the aged group, and 132 lipid droplets in the aged + NMN; PF: *n* = 59 lipid droplets in the young group, 180 lipid droplets in the aged group, and 727 lipid droplets in the aged + NMN group; SF: *n* = 783 lipid droplets in the young group, 558 lipid droplets in the aged group, and 680 lipid droplets in the aged + NMN group; AF: *n* = 2308 lipid droplets in the young group, 453 lipid droplets in the aged group, and 716 lipid droplets in the aged + NMN group). Four mice in each group were sampled. The data are presented as the means ± SEMs. * or #, *p* < 0.05; ** or ##, *p* < 0.01.

To identify the key targets of NMN, we further investigated ovarian ultrastructure by scanning electron microscopy. We studied granulosa cells at different developmental stages in mouse ovarian tissue (Figure 3A) and found that the density of lipid droplets in granulosa cells decreases with age (Figure 3B–E). NMN treatment did not restore the lipid droplet content of PmF‐stage granulosa cells (Figure 3B), but it significantly increased the lipid droplet content of PF‐, SF‐, and AF‐stage granulosa cells (Figure 3C–E). Similarly, the average area of lipid droplets in granulosa cells decreases with age; NMN did not reverse this decrease in PmF‐ or PF‐stage granulosa cells (Figure 3F,G), but it significantly increased the average area of lipid droplets in SF‐ and AF‐stage granulosa cells (Figure 3H,I). These data suggest that NMN may promote the maturation of follicles in the late developmental stages by regulating the properties of energy‐yielding substances, namely, lipid droplets, in granulosa cells at various developmental stages.

**FIGURE 4:**
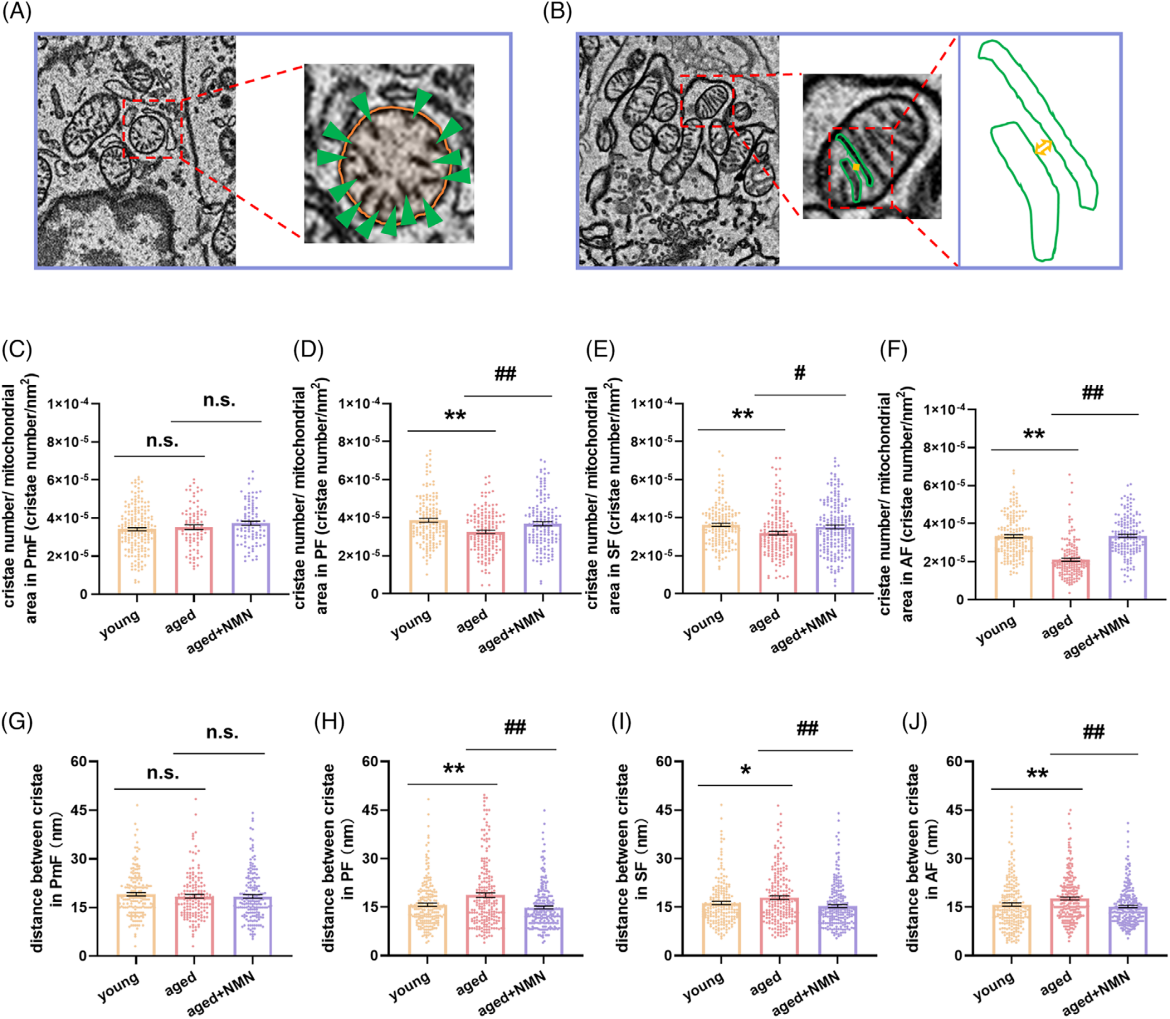
Effects of NMN supplementation on the mitochondrial ultrastructure in mouse granulosa cells. (A) Schematic illustration of the method used to quantify the ratio of the number of cristae to mitochondrial area in mouse granulosa cells. In the enlarged view of the red dashed box, the green arrows indicate cristae within the mitochondria, and the orange circle indicates the mitochondrial area. (B) Schematic illustration of the method used to quantify the intercristal distance in mouse granulosa cells. In the enlarged view of the red dashed box, the green solid line indicates the cristae within the mitochondria, and the yellow bidirectional arrows indicate the distance between adjacent cristae. (C–F) Quantification of cristae number/mitochondrial area in granulosa cells of PmF, PF, SF, and AF from mice in the young, aged, and aged + NMN groups (PmF: *n* = 192 mitochondria in the young group, 86 mitochondria in the aged group, and 94 mitochondria in the aged + NMN group; PF: *n* = 158 mitochondria in the young group, 162 mitochondria in the aged group, and 152 mitochondria in the aged + NMN group; SF: *n* = 164 mitochondria in the young group, *n* = 166 mitochondria in the aged group, and *n* = 202 mitochondria in the aged + NMN group; AF: *n* = 176 mitochondria in the young group, 156 mitochondria in the aged group, and 157 mitochondria in the aged + NMN group). Four mice in each group were sampled. (G–J) Quantification of the intercristae distance in PmF, PF, SF, and AF ovarian granulosa cells of mice in the young, aged, and aged + NMN groups (PmF: *n* = 171 mitochondria in the young group, 48 mitochondria in the aged group, and 171 mitochondria in the aged + NMN group; PF: *n* = 206 mitochondria in the young group, 241 mitochondria in the aged group, and 231 mitochondria in the aged + NMN group; SF: *n* = 194 mitochondria in the young group, 200 mitochondria in the aged group, and 214 mitochondria in the aged + NMN group; AF: *n* = 216 mitochondria in the young group, 233 mitochondria in the aged group, and 229 mitochondria in the aged + NMN group. Four mice in each group were sampled. The data are presented as the means ± SEMs. * or #, *p* < 0.05; ** or ##, *p* < 0.01.

Next, we focused on the mitochondria present in granulosa cells. As shown in Figure 4A, we statistically analyzed the proportion of cristae relative to the mitochondrial area. In PmF‐stage granulosa cells, this parameter did not differ significantly among the three groups of animals (Figure 4C); however, it decreased significantly with age in granulosa cells at other developmental stages (Figure 4D–F), marking a decrease in mitochondrial function as the mitochondrial cristae became sparse. NMN treatment significantly reversed these changes in PF‐, SF‐, and AF‐stage granulosa cells (Figure 4D–F). We then statistically analyzed the intercristal distance within mitochondria in granulosa cells (Figure 4B) and found no significant difference in this parameter in the PmF‐stage granulosa cells present in the three groups (Figure 4G). However, in granulosa cells at other developmental stages, mitochondrial intercristal distance increased with age (Figure 4H–J). NMN treatment significantly alleviated the increase in mitochondrial intercristal distance in PF‐, SF‐, and AF‐stage granulosa cells aging (Figure 4H–J). These data indicate that NMN treatment significantly alters the ultrastructure of mitochondria in granulosa cells, a change that has a positive effect on follicle development and maturation.

### 2.4 Unveiling the mechanism of action of NMN through ovarian transcriptomic analysis

**FIGURE 5:**
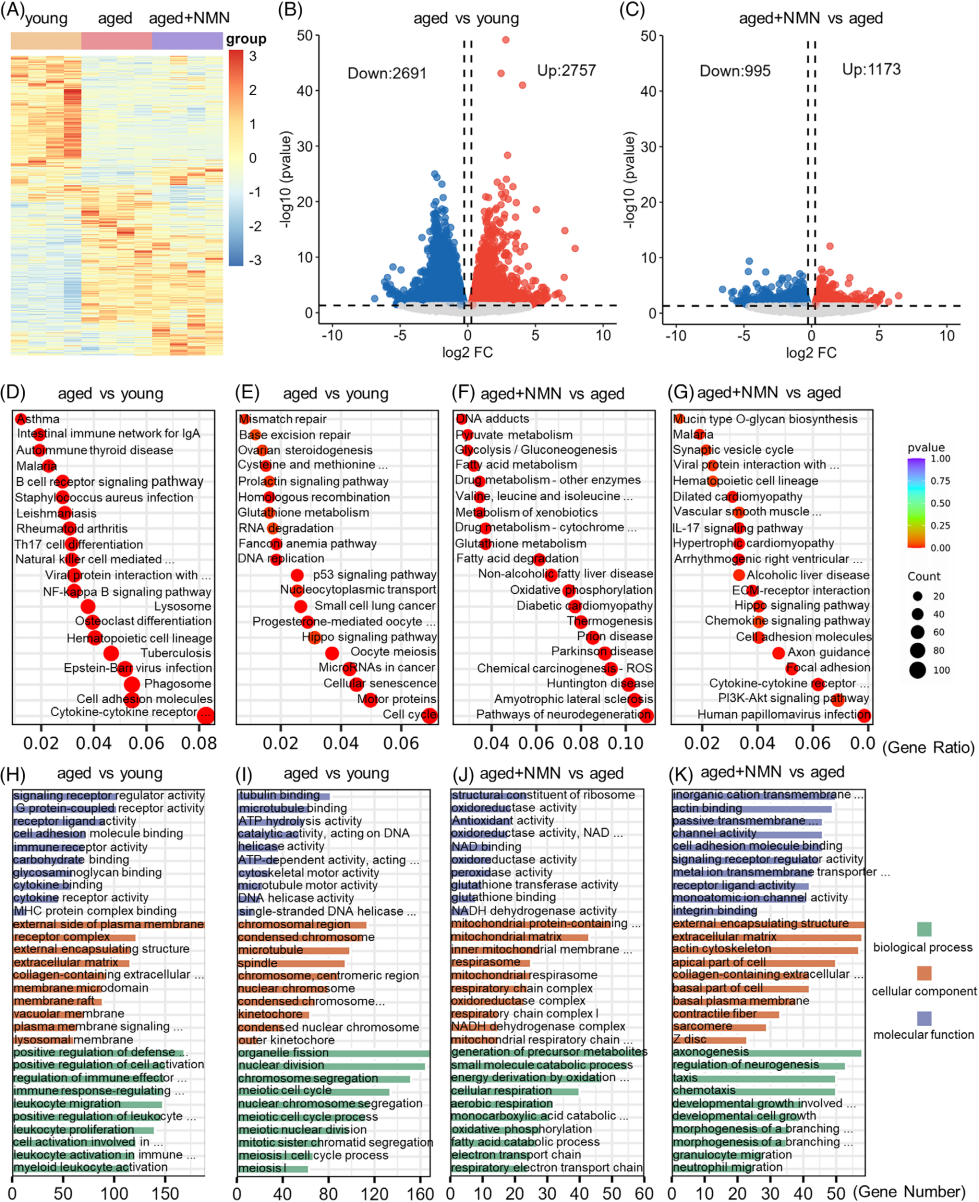
Ovarian transcriptome analysis reveals the mechanism through which NMN inhibits ovarian aging. (A) Heatmap showing the gene expression profiles in the ovaries of young, aged, and aged + NMN groups of mice (*n* = 4 mice per group). (B and C) Volcano plots depicting the differentially expressed genes (DEGs) in aged versus young and aged + NMN versus aged groups of mouse ovaries. Some highly expressed DEGs are listed. Downregulated genes are indicated in blue, and upregulated genes are indicated in red. (D–G) Kyoto Encyclopedia of Genes and Genomes (KEGG) enrichment analysis of upregulated and downregulated DEGs in the ovaries of aged versus young and aged + NMN versus aged mice. (H–K) Gene Ontology (GO) enrichment analysis of upregulated and downregulated DEGs in the ovaries of aged mice versus young mice and aged + NMN mice.

To explore the potential mechanism through which NMN supplementation exerts an antiaging effect on the ovaries of aged mice, we performed transcriptomic analysis of ovarian tissues from young, aged, and aged + NMN mice. Figure 5A presents a heatmap depicting gene expression in the ovaries of mice from the different groups. The heatmap reveals notable differences in gene expression between the young and aged groups. Volcano plot analysis showed that there were 2757 genes that were upregulated and 2691 genes that were downregulated in the aged group compared with the young group (Figure 5B). Compared with the aged group, the aged + NMN group presented 1173 upregulated genes and 995 downregulated genes (Figure 5C).

The differentially expressed genes (DEGs) were subjected to Kyoto Encyclopedia of Genes and Genomes analysis (Figure 5D–G). Compared with the ovaries of the animals in the young group, the ovaries of the aged animals exhibited increased expression of genes associated with immune and inflammatory cell pathways, including genes that encode proteins involved in the NF‐κB signaling pathway, Th17 cell differentiation, *Staphylococcus aureus* infection, the B‐cell receptor signaling pathway, and natural killer cell‐mediated cytotoxicity. Moreover, genes related to intracellular structure and function, cell‒cell interactions, and signal transduction (such as genes that encode proteins associated with lysosomes, phagosomes, cell adhesion molecules, and cytokine receptor interactions) were also upregulated. The upregulation of these biological pathways reflects increased activity of the immune system, regulation of intracellular structure and function, and changes in cell‒cell interactions and signal transduction in aged mouse ovarian tissues and suggest that pathways associated with inflammation and immune reactivity are relatively more active in aged mouse ovaries than in the ovaries of young mice (Figure 5D). Conversely, genes associated with DNA damage repair (including mismatch repair and base excision repair), cell signaling (the prolactin, P53, and hippo signaling pathways), the cell cycle (DNA replication, luteal progesterone‐mediated oocyte maturation, homologous recombination, and embryonic meiosis), and metabolism‐related pathways (ovarian steroidogenesis and cysteine and methionine metabolism) were downregulated in aged mice, indicating a functional decline in DNA repair, cell signaling, cell proliferation, and metabolic processes in the ovarian tissues of aged mice (Figure 5E).

NMN treatment led to the upregulation of genes associated with energy‐related metabolic pathways such as oxidative phosphorylation, pyruvate metabolism, fatty acid metabolism, GSH metabolism, glycolysis/gluconeogenesis, fatty acid degradation, and valine, leucine, and isoleucine degradation in the ovaries of the mice in the aged group (Figure 5F), indicating an increase in energy‐related metabolic processes such as amino acid metabolism, lipid metabolism, and oxidative phosphorylation in aged mouse ovarian tissues following NMN treatment. Conversely, genes associated with inflammation and immunity‐related pathways (cytokine receptor interactions, the chemokine signaling pathway, and the IL‐17 signaling pathway), cell‒cell interactions, cell structure‐related pathways (cell adhesion molecules, focal adhesion, and the ECM‒receptor interaction), and signal transduction pathways (the Hippo signaling pathway and the PI3‒Akt signaling pathway) were downregulated (Figure 5G), indicating that NMN treatment can alleviate inflammatory responses in the ovarian tissues of aged mice and that it can regulate processes such as cell adhesion, migration, and extracellular matrix remodeling, thus affecting physiological processes such as cell growth and proliferation.

Gene Ontology analysis of the DEGs revealed enhanced immune responses and signaling, inhibition of DNA repair, cytoskeleton reconstruction, and cell proliferation processes in the ovaries of aged mice, whereas NMN treatment promoted mitochondrial function and energy‐related metabolic processes, ultimately resulting in the maintenance of normal cellular physiological functions (Figure 5H–K). Overall, NMN treatment enhances mitochondrial function and cellular energy metabolism while also mitigating ovarian inflammation by modulating the relevant pathways and cellular interactions. NMN treatment may thus influence cellular function and inflammatory status, providing potential mechanisms for the promotion of cellular health and anti‐inflammatory processes.

### 2.5 Validation of the transcriptomic analysis results via immunoblotting

**FIGURE 6:**
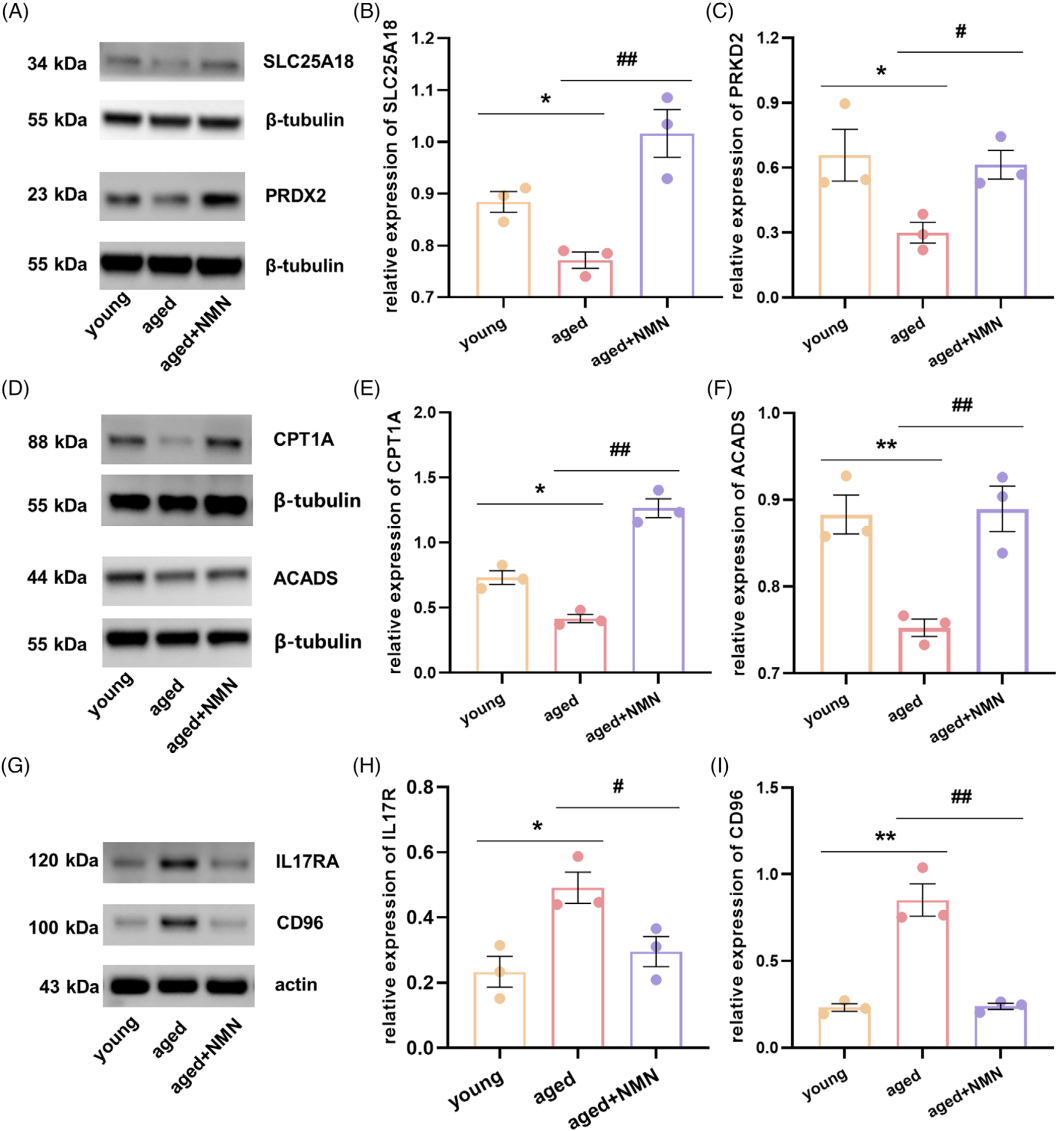
Validation of the transcriptome analysis results through protein immunoblotting. (A) Immunoblot images for the mitochondrial function‐related proteins solute carrier family 25 member 18 (SLC25A18) and peroxiredoxin 2 (PRDX2). (B and C) Changes in the expression of the mitochondrial function‐related genes SLC25A18 and PRDX2 in the ovaries of young, aged, and aged + NMN mice (*n* = 3 mice per group). (D) Immunoblot images for the fatty acid oxidation‐related proteins carnitine palmitoyltransferase 1A (CPT1a) and acyl‐CoA dehydrogenase short chain (ACADS). (E and F) Changes in the expression of the fatty acid oxidation‐related genes CPT1A and ACADS in the ovaries of young, aged, and aged + NMN mice (*n* = 3 mice per group). (G) Immunoblot images for the inflammation‐related proteins interleukin‐17 receptor A (IL17RA) and CD96. (H and I) Changes in the expression of the inflammation‐related genes IL17RA and CD96 in the ovaries of young, aged, and aged + NMN mice (*n* = 3 mice per group). The data are presented as the means ± SEMs. * or #, *p* < 0.05; ** or ##, *p* < 0.01.

From the DEGs identified in the ovarian transcriptome analysis, we selected DEGs that are related to mitochondrial function, energy metabolism, and inflammatory and immune pathways. We conducted a statistical analysis of the transcriptomic data for these genes; the results are presented in Figure S3. Next, we employed immunoblotting to validate the expression of DEGs that were identified in the transcriptomic data. As depicted in Figure 6A–C, the expression of genes related to mitochondrial function, solute carrier family 25 member 18 (SLC25A18) and peroxiredoxin 2 (PRDX2), was much lower in the aged group than in the young group. However, following NMN treatment, the expression of these proteins in the aged group markedly increased, suggesting that NMN can ameliorate age‐related ovarian mitochondrial dysfunction. As shown in Figure 6D–F, we subsequently analyzed the expression of the genes encoding carnitine palmitoyltransferase 1A (CPT1a) and acyl‐CoA dehydrogenase short chain (ACADS), both of which are linked to fatty acid β‐oxidation. Factors linked to ovarian fatty acid β‐oxidation are downregulated with age, whereas NMN treatment significantly increases the expression of these genes in the ovaries of aged mice, promoting lipid droplet degradation. Since fat serves as a crucial source of energy for follicle development, enhancing fat metabolism is essential for follicular development and maturation. Finally, as illustrated in Figure 6G–I, we validated the expression of proteins linked to inflammation in the ovaries of different groups of mice. The expression levels of the proteins encoded by the inflammation‐related genes IL‐17 receptor A (IL17RA) and CD96 in ovarian tissue were considerably greater in the aged group than in the young group. The expression levels of these proteins in the ovaries of the mice in the aged group, however, decreased dramatically following NMN administration.

### 2.6 Long‐term NMN supplementation improves ovarian health in aging mice

**FIGURE 7:**
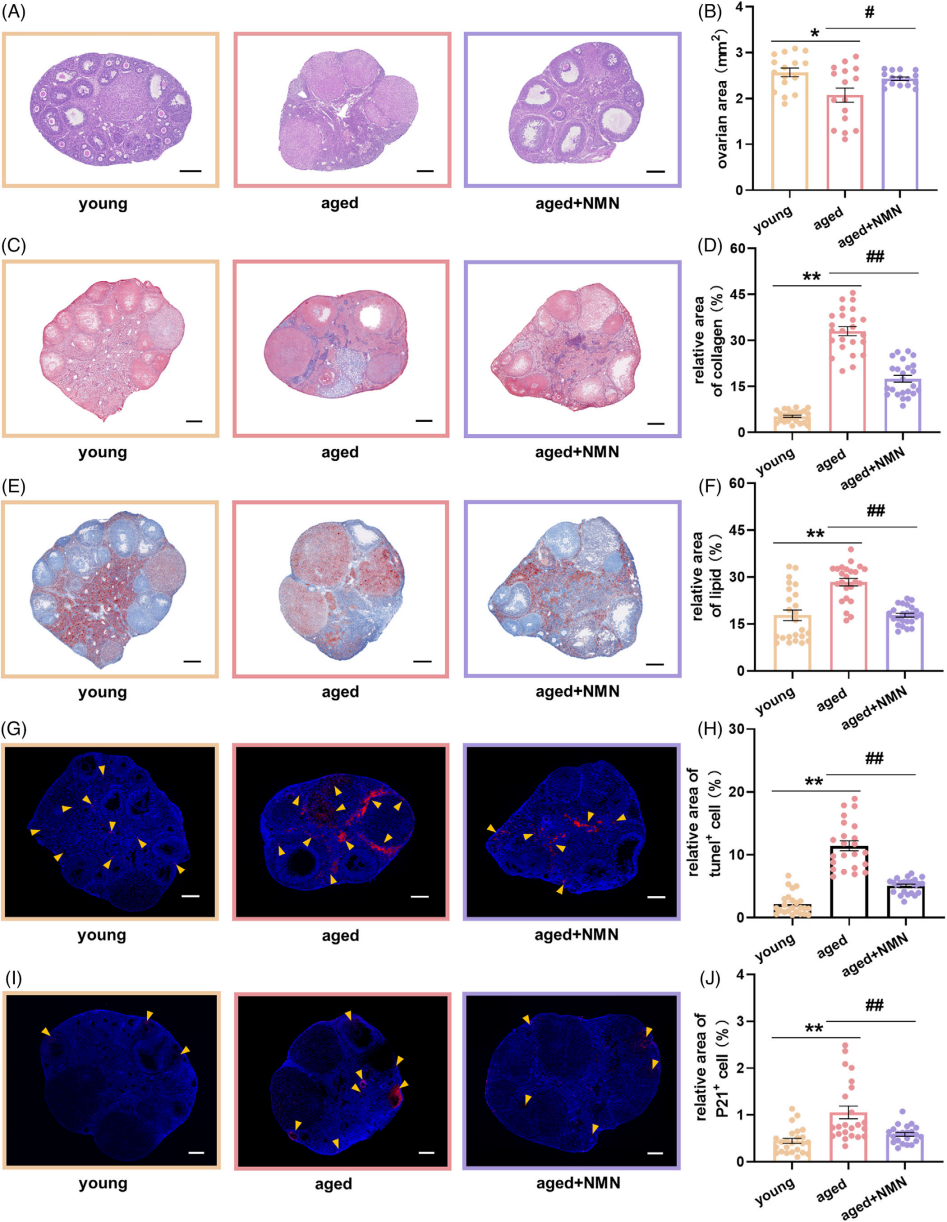
Supplementation with NMN improves the health of aged ovaries. (A) Representative images of HE‐stained sections of ovaries from mice in the young, aged, and aged + NMN groups. Scale bar, 200 µm. (B) Statistical analysis of the area of ovarian sections from different groups (*n* = 16 sections per group from 4 mice). (C) Representative images of Masson's‐stained ovarian sections from mice in the young, aged, and aged + NMN groups. Scale bar, 200 µm. (D) Statistical analysis of the area of collagen‐positive regions in ovarian sections from different groups (*n* = 24 sections per group from 4 mice). (E) Representative images of Oil Red O‐stained sections of mouse ovaries from young, aged, and aged + NMN groups. Scale bar, 200 µm. (F) Statistical analysis of the area occupied by lipid components in ovarian sections from different groups (*n* = 24 sections per group from 4 mice). (G) Representative images of TUNEL‐stained ovarian sections from mice in the young, aged, and aged + NMN groups. Blue: DAPI staining. Yellow arrows indicate TUNEL‐positive signals. Scale bar, 200 µm. (H) Statistical analysis of the area of TUNEL‐positive regions in ovarian sections from different groups (*n* = 24 sections from young mice and 23 sections from aged and aged + NMN mice (4 mice from each group)). (I) Representative images showing P21 protein immunohistochemistry in ovarian sections from mice in the young, aged, and aged + NMN groups. Blue: DAPI staining. Yellow arrows indicate P21‐positive signals. Scale bar, 200 µm. (J) Statistical analysis of the area of P21‐positive regions in ovarian sections from different groups (*n* = 24 sections from young mice, 23 sections from aged mice, and 22 sections from aged + NMN mice (4 mice from each group)). The data are presented as the means ± SEMs. * or #, *p* < 0.05; ** or ##, *p* < 0.01.

We specifically designed experiments to verify the role and mechanism of action of NMN in reversing ovarian aging. Frozen sections of ovarian tissue were stained with HE (Figure 7A). Statistical analysis of the area of the ovarian sections revealed that aging significantly reduced the area of the mouse ovarian sections and that NMN treatment effectively reversed ovarian atrophy (Figure 7B). Masson's trichrome staining of ovarian sections (Figure 7C) revealed that while the area occupied by collagen‐positive regions in mouse ovaries increased significantly with age, the area occupied by collagen‐positive regions decreased dramatically after NMN treatment (Figure 7D). Ovarian sections prepared from the animals in the different groups were also stained with Oil Red O (Figure 7E). The staining revealed that, in the young group, lipids were predominantly distributed in the ovarian stromal region. In contrast, aging tended to increase lipid accumulation in ovarian parenchymal cells. After NMN treatment, statistical analysis of the area occupied by lipid components in ovarian sections from the different groups revealed that aging increased the area occupied by lipid components, whereas NMN treatment of aged mice significantly reduced it (Figure 7F). These findings suggest that NMN treatment reverses the changes that otherwise occur in the tissue composition of aging mouse ovaries and that it inhibits fibrosis, resulting in a more normal distribution of lipid components in the tissue.

TUNEL staining of ovarian sections prepared from the mice in the different groups was performed to detect apoptosis (Figure 7G). Compared with the young group, the aged group presented more TUNEL‐positive signals and larger puncta, whereas after NMN treatment, the cellular area that showed TUNEL‐positive signals significantly decreased (Figure 7H). These data indicate that NMN can inhibit increased apoptosis in aging ovaries. Immunohistochemical staining of ovarian sections for the expression of the aging marker protein P21 (Figure 7I) revealed that aging led to a significantly larger area of P21‐positive cells in the young group than in the aged group. After NMN treatment, the area occupied by P21‐positive cells decreased significantly (Figure 7J). These data indicate that NMN treatment can inhibit the expression of markers of aging and reverse ovarian aging.

**FIGURE 8:**
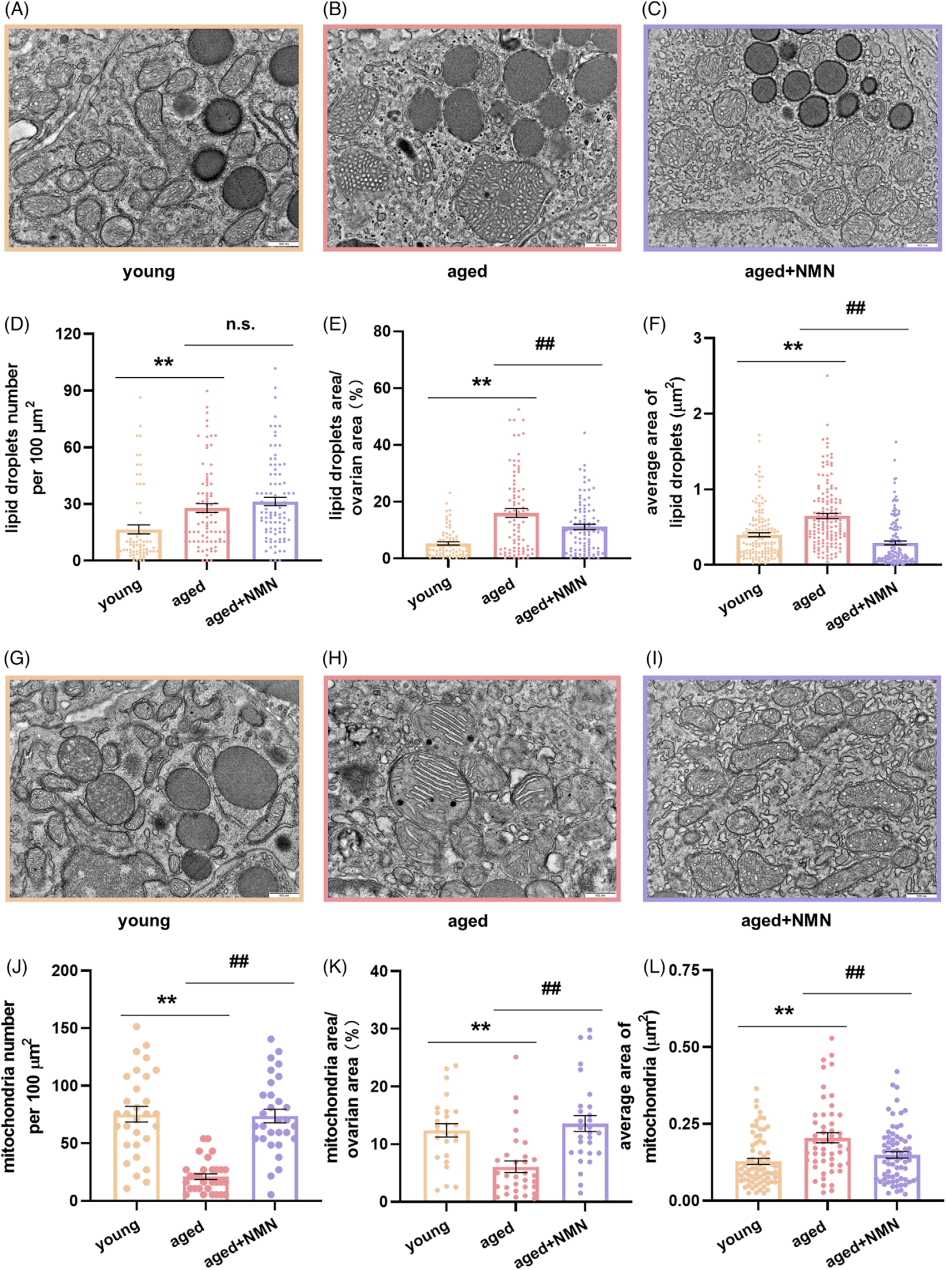
Effect of NMN supplementation on lipid droplets and mitochondria within ovaries. (A–C) Representative images showing lipid droplets in the ovaries of the mice in the young, aged, and aged + NMN groups. Scale bar, 500 µm. (D–F) Statistical analysis of the number of lipid droplets per unit area, the ratio of lipid droplet area to ovarian area (*n* = 72 figures from young mice, 84 figures from aged mice, and 95 figures from aged + NMN mice,4 mice from each group were analyzed), and the average area occupied by lipid droplets (*n* = 147 lipid droplets per group from 4 mice) in electron microscopy images. (G–I) Representative electron microscopy images of mitochondria in the ovaries of young, aged, and aged + NMN groups. Scale bar, 500 µm. (J–L) Statistical analysis of the number of mitochondria per unit area and the ratio of total mitochondrial area to ovarian area (*n* = 72 figures from the mice in the young group, 84 figures from the mice in the aged group, and 95 figures from the mice in the aged + NMN group; 4 mice from each group were analyzed) and the average area occupied by mitochondria (*n* = 72 mitochondria from the young group, 51 mitochondria from the aged group, and 70 mitochondria from the aged + NMN group; 4 mice from each group were analyzed) in electron microscopy images from different groups. The data are presented as the means ± SEMs. ** or ##, *p* < 0.01.

Next, we used electron microscopy to observe and analyze mitochondria (Figure 8A–C) and lipid droplets (Figure 8G–I) in mouse ovarian tissues. We found that aging significantly reduced the number of mitochondria per unit area (Figure 8D) and the ratio of mitochondrial area to ovarian area (Figure 8E) and that it significantly increased the average area occupied by mitochondria (Figure 8F) in mouse ovarian tissues. NMN treatment effectively reversed these changes in the mitochondria of aging ovaries. Analysis of ovarian lipid droplets revealed that aging led to a significant increase in the number of lipid droplets per unit area, the ratio of lipid droplet area to ovarian area, and the average size of the lipid droplets. While NMN treatment had no effect on the number of lipid droplets per unit area (Figure 8J), it significantly reduced the average size of the lipid droplets (Figure 8K) and the ratio of lipid droplet area to ovarian area (Figure 8L). These data suggest that NMN may reverse ovarian aging by regulating mitochondrial function and lipid metabolism.

## 3 DISCUSSION

In today's fast‐paced lifestyle, the issue of fertility in older women is becoming increasingly prominent; this has resulted in a general decline in fertility that is manifested by increased incidences of ovulatory disorders, embryonic chromosomal abnormalities, and miscarriages, leading to a decrease in pregnancy rates. Even with the help of ARTs, the diminished ability to produce enough high‐quality oocytes remains a key limiting factor for the fertility of older women.^3^, ^6^, ^35^ Safe and effective strategies to reverse age‐related ovarian infertility have not yet been identified. NAD^+^ is an essential oxidoreduction cofactor and enzyme substrate that is involved in regulating cellular processes such as energy metabolism, oxidative damage, and inflammatory responses. Tissue NAD^+^ levels decrease with age and are closely associated with aging.^36^, ^37^, ^38^, ^39^

NMN is an important precursor substance for NAD^+^ synthesis in the body. This study revealed that NMN supplementation can reverse the decrease in NAD^+^ levels that normally occurs in ovarian tissues due to aging and that it effectively inhibits ovarian atrophy in aged mice. Ovarian atrophy can lead to disorders in ovarian hormone secretion, and NMN treatment can significantly increase the concentrations of E2, AMH and FSH in the serum of aged mice, thereby promoting ovarian function.^40^, ^41^ Inhibition of ovarian atrophy creates favorable conditions for the development of oocytes and embryos.^42^, ^43^ Our research further indicated that supplementation with NMN can reverse the age‐related decline in the ovulation rate and reduce the proportion of abnormal oocytes, consistent with previous reports in the literature.^32^, ^44^, ^45^ Analysis of ovarian reserves in aged mice subjected to different treatments revealed a significant decrease in the quantity of follicles at various developmental stages. However, NMN treatment significantly increased the quantity of follicles at various phases of development and effectively inhibited follicular atresia, indicating that it can play a positive role in slowing the decline in ovarian reserves due to aging. Nonetheless, experiments on mouse reproductive capacity demonstrated that NMN treatment of aged mice did not improve their fertility (data not shown); this may be related to the advanced age of the animals or to extremely low fertility or loss of fertility.

Our study of mitochondrial distribution and membrane potential in ovulation‐induced oocytes using mitochondrial dyes revealed that NMN treatment can inhibit the clustered distribution pattern of mitochondria in oocytes caused by aging and that it can increase the mitochondrial membrane potential, thereby enhancing mitochondrial function. Moreover, supplementation with NMN effectively reduced the level of ROS in the oocytes of aged mice. Similar beneficial changes have been reported in previous studies.^32^, ^44^ Our measurement of the serum levels of the antioxidant factors GSH and MDA in the mice in the different groups further validated the above findings; namely, NMN treatment reduces the degree of oxidative stress in the body. The improvements in mitochondrial function and reductions in ROS levels that occur after NMN treatment positively affect the body's health status and are accompanied by considerable decreases in the levels of expression of the proinflammatory factors IL‐1β and TNF‐α in the serum.

We focused particularly on the effects of NMN on lipid droplets and mitochondria in the granulosa cells of follicles. Granulosa cells provide essential nutrients and hormonal support to oocytes, and their health directly impacts oocyte maturation and quality.^32^, ^33^ Lipid droplets and mitochondria play key roles in cellular energy metabolism and in the stress response. Studies have shown that regulating lipid droplets and improving mitochondrial function in granulosa cells can increase the energy supply and the antioxidant defense capabilities of oocytes, thereby increasing their capacity for growth and viability.^34^, ^35^ We also investigated whether treatment with NMN affects the ultrastructure of ovaries by scanning electron microscopy. Studies have revealed that ATP produced through the mitochondrial metabolism of fatty acids present in lipid droplets is an important source of energy for follicle and embryo development.^21^, ^46^ We observed a decrease in the number of lipid droplets and in the average area occupied by lipid droplets in the granulosa cells of mouse follicles with age. However, after NMN treatment, we noted an increase in both the size and the content of lipid droplets in granulosa cells, especially in granulosa cells at the SF and AF stages. These findings reflect the fact that energy is needed for follicle development and maturation. Mitochondria are an essential factor in follicle and embryo development.^7^, ^47^, ^48^, ^49^ We found that the ratio of the number of mitochondrial cristae to mitochondrial area in granulosa cells at the PF, SF, and AF stages decreased with age, whereas the spacing between mitochondrial cristae increased with age, indicating that the mitochondrial cristae became sparse and that mitochondrial function declined in aged animals. NMN supplementation effectively inhibited the age‐related sparse distribution of mitochondrial cristae, thus enhancing mitochondrial function. The 8‐week NMN treatment had positive effects on cells at the SF and AF stages, whereas no similar significant effects were observed in cells at the PmF or PF stages, possibly because of the insufficient duration of the NMN treatment. The significant increase in the number of follicles at the PmF and PF stages following NMN treatment might be due to promotion by NMN of other aspects of ovarian health, leading to these beneficial outcomes.

To further elucidate the mechanism by which NMN reverses ovarian aging and improves oocyte quality, we conducted transcriptome sequencing analysis of ovarian tissues from mice subjected to different treatments. The results showed that NMN treatment significantly increased energy‐related metabolic activity and enhanced biological processes and pathways related to mitochondria while alleviating ovarian inflammation. We validated this by immunoblotting. SLC25A18, a mitochondrial transporter protein, is responsible for transporting glutamate from the cytoplasm to the mitochondrial matrix, where glutamate participates in metabolic reactions.^50^, ^51^ PRDX2 is an antioxidant enzyme that is essential for preserving redox balance and extending cell lifespan.^52^ The immunoblotting results revealed that NMN effectively reversed the age‐associated decrease in the expression of the mitochondrial function‐related proteins SLC25A18 and PRDX2.

CPT1A and CPT2 catalyze the entry of free fatty acids generated by lipid droplet oxidation into the mitochondrial matrix.^53^, ^54^ In addition, members of the acyl‐CoA dehydrogenase family, such as ACADS, catalyze the initial reaction in the mitochondrial fatty acid β‐oxidation pathway.^55^ Our findings revealed that NMN treatment dramatically increased the expression of the CPT1A and ACADS genes, which are involved in mitochondrial β‐oxidation, in the ovaries of aged mice, indicating that NMN enhances the capacity for mitochondrial lipid metabolism. IL‐17RA is the receptor for IL‐17A, which protects cells from invasion by pathogens and promotes pathological inflammation in autoimmune diseases.^56^ CD96 is a receptor belonging to the immunoglobulin superfamily and is involved in inflammatory responses.^57^, ^58^ With age, the protein expression of IL7RA and CD96 increases significantly, but NMN treatment can significantly reduce the expression of these proteins in ovarian tissue.

To verify the proposed mechanism of action of NMN, we studied long‐term NMN supplementation and its impact on ovarian health and discovered that NMN helps prevent ovarian atrophy. The degree of ovarian fibrosis is related to the degree of inflammation, as demonstrated by Masson's trichrome staining, which revealed that aging leads to excessive production and accumulation of the extracellular matrix in mouse ovaries, thereby increasing the amount of connective tissue and exacerbating fibrosis.^59^ NMN treatment effectively reversed these fibrotic changes in the ovaries of aging mice. Analysis of Oil Red O‐stained sections revealed that, in mice in the young group, lipids were predominantly located in the ovarian stromal region; however, aging caused a more pronounced accumulation of lipids in ovarian parenchymal cells. This shift in lipid distribution may be closely linked to ovarian aging.^60^ Long‐term NMN treatment improved the lipid droplet content of aged mouse ovaries.

Studies have reported that ATP produced through the mitochondrial oxidation of fatty acids from lipid droplets is an important source of energy for follicle and embryo development.^21^, ^46^ Through observation and statistical analysis of mitochondrial‐related parameters at the ovarian level, we noted that aging results in larger mitochondrial size and decreased numbers of mitochondria. This may be associated with decreased mitochondrial fission and fusion.^7^, ^61^ NMN treatment may increase these activities. Additionally, our analysis of lipid droplets at the ovarian level revealed that NMN treatment reduces the average area of lipid droplets in the ovaries of aged mice, thereby decreasing the density at which ovarian lipid droplets are distributed. On the basis of our statistical analysis of lipid droplets at the granulosa cell level, we can conclude that aging leads to a decrease in the lipid droplet content of granulosa cells, whereas the lipid droplet content of nongranulosa cells increases as the overall lipid droplet content of aging ovaries increases. Our results indicate that NMN treatment improves the distribution of lipid droplets in aging ovaries as well as the lipid droplet content of granulosa cells. Enhanced mitochondrial dynamics and proper lipid distribution and metabolism help reduce lipid accumulation, lower oxidative stress and inflammation levels, and provide favorable conditions for follicular development.^4^, ^62^, ^63^ Long‐term NMN treatment also significantly reduced the proportions of apoptotic signals and markers of aging in the ovaries of aged mice. These results imply that NMN treatment supports normal reproductive and endocrine functions in aging ovaries.

Although supplementation with NMN has shown potential benefits in improving mitochondrial function, enhancing energy metabolism, and reducing ovarian inflammation, our findings should be validated in a larger population to ensure their universality and reproducibility. To date, we have not conducted ART‐related research or clinical trials involving NMN treatment, but these studies are both necessary and significant and warrant more attention and research in the future. Furthermore, to deepen our understanding of how NMN specifically regulates related molecular mechanisms, future studies could employ advanced technologies such as single‐cell sequencing and spatial transcriptomics. These technologies not only can reveal cellular heterogeneity but can also provide detailed information about intercellular interactions, thereby offering a more solid scientific basis for the clinical application of NMN in enhancing the fertility of older women.

Our study suggests that supplementation with NMN enhances cellular mitochondrial function and energy metabolism. NMN also reduces ovarian inflammation levels by modulating pathways related to inflammation and immunity, as well as cell‒cell interactions. These changes collectively improve the health status of aging ovaries and increase the number and quality of ovulated oocytes, providing a potentially effective strategy for increasing fertility in older women and obtaining more high‐quality oocytes for ART.

## 4 METHODS

### 4.1 Mice

Approval for the experimental project was obtained from the Animal Ethics Committee of Peking Union Medical College Hospital. The National Institutes of Health and the Animal Welfare Act have established rules for the care and use of laboratory animals, and these rules were followed during the experimental procedures. Two‐month‐old (young) and 12‐month‐old (aged) female C57/BL6J mice were procured from the Beijing Vital River Experimental Animal Center (Beijing, China). The mice were specifically pathogen‐free and were kept in rooms that were maintained between 20 and 25°C under a 12‐h light/dark cycle. Throughout the entire study period, the mice had unlimited access to water and food. NMN (Jin'an Yunqiao Health Management Co., Ltd., Shanghai, China.) was delivered intraperitoneally to the animals in the aged + NMN group at a dose of 500 mg/kg body weight; the animals in the young and aged groups received the same amount of physiological saline. The injections were administered consecutively for a duration of 10 days. In long‐term NMN treatment experiments, the aged + NMN group received continuous administration of NMN through the drinking water at a concentration of 2 g/L for 8 weeks.

### 4.2 Determination of ovarian NAD^+^ content

To measure NAD^+^ levels, ovarian samples were analyzed using the NAD/NADH Quantification Kit (Sigma‒Aldrich; cat# MAK037) according to the manufacturer's instructions. Briefly, ovaries were collected from the mice, weighed, homogenized in lysis buffer, and centrifuged, and the supernatant was collected for subsequent analysis. Using a colorimetric assay set at 450 nm, the amounts of total NAD (NAD^+^ and NADH) and NADH were determined. The NAD^+^ concentration was obtained by subtracting the NADH value from the total NAD concentration.

### 4.3 Serum analysis

Mouse blood samples were collected via eye enucleation and centrifuged to obtain the supernatant. Enzyme‐linked immunosorbent assay kits were used to measure the concentrations of the following chemicals in the serum: E2 (ABclonal; cat# RK00651), AMH (ABclonal; cat# RK09261), FSH (ABclonal; cat# RK04237), GSH (ABclonal; cat# RK04298), MDA (ABclonal; cat# RK04090701), IL‐1β (ABclonal; cat# RK04381), and TNF‐α (ABclonal; cat# RK04381).

### 4.4 Ovarian sectioning and analysis

In long‐term NMN treatment experiments, dehydrated mouse ovaries designated for follicle number analysis were embedded in cold embedding medium (SAKURA; cat# 4583) and then cut into 8‐µm‐thick sections using a cryostat. Every 10th section was collected for HE staining and analysis. In subsequent validation experiments, the embedded tissues were sliced at a thickness of 8 µm using a cryostat, and every 10th section was collected for HE staining and ovarian area measurement. Every 11th section was collected for Masson's staining analysis, every 12th section was collected for Oil Red O staining analysis, every 13th section was collected for TUNEL staining analysis, and every 14th section was collected for immunohistochemical staining analysis of P21. The collected tissue sections were stained using the following kits: HE staining kit (Solarbio; G1120), Masson's trichrome staining kit (Solarbio; G1340), Oil Red O staining kit (Beyotime; C0157M), and TUNEL assay apoptosis detection kit (Tiosbio; T6039).

### 4.5 Immunohistochemistry

Frozen sections were obtained for immunohistochemistry as described above. After fixation in 4% paraformaldehyde for 30 min, the sections were washed three times with PBS (pH = 7.4). Three PBS washes were conducted after antigen retrieval using EDTA antigen retrieval buffer (SantaiBio; GC‐CW0129). The sections were then blocked with 5% BSA for 30 min and incubated with an antibody against P21 (ABclonal; A19094) overnight at 4°C. Following three washes with PBST solution, the secondary antibody (ABclonal; AS058) was added, and the samples were incubated for 30 min at 37°C. After three washes with PBST solution, DAPI staining solution was added to the sections, and the sections were incubated for 10 min at room temperature.

### 4.6 Electron microscopy

Mouse ovarian tissues were submerged in phosphate‐buffered saline (0.1 M, pH 7.4) containing 4% (w/v) paraformaldehyde and 2.5% glutaraldehyde (Sigma; G5886) and fixed overnight at 4°C. The ovarian tissue blocks were subsequently submerged in 0.1 M phosphate‐buffered saline containing 2% osmium tetroxide (Ted Pella; 18451) at room temperature for 90 min followed by submersion in 0.1 M phosphate‐buffered saline containing 2.5% ferrocyanide (Sigma; 234125) for 90 min. After three washes with 0.1 M phosphate‐buffered saline, the samples were incubated for 45 min at 40°C with filtered thiocarbohydrazide (Sigma; 223220). The tissue blocks were then fixed again with 2% osmium tetroxide for 90 min and incubated overnight at 4°C in an aqueous solution of 1% uranyl acetate. After dehydration by sequential passage through 50, 70, 80, 90, and 100% ethanol and finally pure acetone (10 min each time), the tissue blocks were embedded in Epon 812 resin (SPI; 02660‐AB). A diamond knife was used to cut ultrathin slices, and the samples were imaged on a Zeiss Gemini 300 scanning electron microscope. ImageJ software was used to analyze the electron microscopy images of mouse ovarian follicles at various developmental stages. Lipid droplet density—defined as the ratio of the lipid droplet area to the granulosa cell area—was quantified, as was the average area of individual lipid droplets, to assess the energy‐related metabolic activity of granulosa cells. The ratio of the number of mitochondrial cristae to the mitochondrial area, as well as the spacing between the cristae, were also measured to permit evaluation of the structural characteristics of the mitochondria. In the measurement of the entire ovary, lipid droplet or mitochondrial density was defined as the number of lipid droplets or mitochondria per unit area, as well as the ratio of the lipid droplet or mitochondrial area to the total ovarian area. Additionally, the average area of lipid droplets and mitochondria was calculated. These parameters were used to assess energy‐related metabolic activity at the whole‐ovary level.

### 4.7 Collection of oocytes

To induce ovulation, 10 IU of pregnant mare serum gonadotropin (Jianchun, Nanjing, China; cat# A006) was intraperitoneally administered to female mice. Forty‐eight hours later, 7.5 IU of human chorionic gonadotropin (hCG; Jianchun; cat# A001‐2) was injected. Cumulus–oocyte complexes (COCs) were isolated from the oviduct ampulla 13.5 h after the hCG injection and placed in M2 medium (Sigma‒Aldrich; cat# M7167). To eliminate cumulus cells, the COCs were briefly treated with 1 mg/ml hyaluronidase in M2 medium.

### 4.8 Confocal microscopy

To analyze the mitochondrial distribution pattern, oocyte samples were incubated in M16 medium (Thermo Fisher Scientific; cat# M7512) containing 500 nM MitoTracker Red at 37°C for 30 min in a dark environment. The oocytes were then rinsed several times in M2 medium, placed in a glass‐bottom culture dish, and examined via a laser scanning confocal microscope.

The mitochondrial membrane potential was assessed using the MitoProbe JC‐1 Assay Kit (Thermo Fisher Scientific; cat# M34152). Oocytes were incubated in M16 medium containing 2 µM JC‐1 for 30 min at 37°C in a dark environment and then washed with PBS. As soon as possible, the oocytes were moved to a culture dish with a glass bottom and examined in a laser scanning confocal microscope. The red‐to‐green fluorescence intensity ratio represents the mitochondrial membrane potential (ΔΨm). The amount of ROS in the oocytes was determined using an ROS assay kit (Jianchun; cat# E004). Oocytes were incubated with 10 µM DCFH diacetate for 30 min at 37°C in DPBS containing 0.1% BSA. Following a DPBS wash, the oocytes were placed in a culture dish with a glass bottom for observation. Confocal microscope images were analyzed via ImageJ software.

### 4.9 Immunoblotting analysis

Mouse ovarian tissues were homogenized in standard lysis buffer. Proteins were electrophoresed on 8% and 15% Bis–Tris precast gels and transferred to PVDF membranes. The membranes were blocked for 1 h at room temperature with 5% skim milk and then incubated with the corresponding primary antibodies overnight at 4°C. After six TBST washes, the membranes were treated with the appropriate secondary antibodies coupled to horseradish peroxidase for 1 h. The following antibodies were used: anti‐Slc25a18 (proteintech; cat# 17348‐1‐AP), anti‐PRDX2 (Cell Signaling Technology (CST); cat# 46855S), anti‐IL17RA (ABclonal; cat# A5163), anti‐CD96 (ABclonal; cat# A20547), anti‐CPT1a (proteintech; cat# 15184‐1‐AP), anti‐ACADS (ABclonal; cat# A20926), anti‐beta‐tubulin (ABclonal; cat# A12289), and anti‐β‐actin (ABclonal; cat# AC004). Visualization of the immunoreactive bands was performed via the ECL Super Kit (ABclonal; cat# RM02867), and the immunoreactive bands were analyzed using ImageJ software.

### 4.10 Ovarian RNA library construction and transcriptome sequencing

From each group of animals, four sets of samples were collected, with two ovaries per sample, and preserved in lysis buffer. RNase inhibitor and the components of the cell lysis buffer were present in the cell collecting fluid. Reverse transcription was performed using the nucleotide sequence of oligo‐dT to generate the first cDNA. The first cDNA was then subjected to PCR amplification to enrich nucleic acids. The amplification products were purified, and a library was constructed; construction of the library involved DNA fragmentation, end repair, addition of “A” and adapters, PCR amplification, and library quality control.

The constructed library was sequenced on the Illumina NovaSeq 6000 platform using the PE150 sequencing method. The original downstream sequences (raw reads) obtained from HiSeq sequencing were subjected to a process that removed low‐quality sequences and connector contamination. Throughout this processing, high‐quality sequences (clean reads) were generated, and these reads served as the foundation for all further investigations.

### 4.11 Quantification and statistical analysis

The mean ± SEM was used to report percentage or numerical data obtained from at least three separate replicates. Student's *t*‐tests applied through the statistical program GraphPad Prism 8 were used for statistical analysis. *p* < 0.05 was considered statistically significant.

## AUTHOR CONTRIBUTIONS

Rong Chen, Peng Zhang, and Jinghui Liang designed the research. Jinghui Liang and Feiling Huang performed the experiments and analyzed results. Xueyu Hao conducted the analysis of the transcriptome data. Rong Chen, Peng Zhang, and Jinghui Liang wrote and prepared this manuscript. All authors have read and approved the final manuscript.

## CONFLICT OF INTEREST STATEMENT

The authors declare no conflict of interest.

## ETHICS STATEMENT

The animal experimental procedures were approved by the Welfare and Ethical Committee of Peking Union Medical College Hospital (XHDW‐2022‐147).

## Supporting information

Supporting Information

## Data Availability

The RNA‐seq data can be obtained from the GSA‐human database (https://ngdc.cncb.ac.cn/gsa‐human/) with accession number: CRA017492. The processed data and analysis codes are available upon reasonable request from the corresponding author. Other data can be obtained from the corresponding author upon justifiable inquiries.
