# Supplementary material for: Nicotinamide mononucleotide supplementation rescues mitochondrial and energy metabolism functions and ameliorates inflammatory states in the ovaries of aging mice
Source: MedComm (2020). 2024 Sep 30;5(10):e727. doi: 10.1002/mco2.727 (PMC11442848; doi:10.1002/mco2.727)
Supplement: Supplementary file 1 — Supporting Information [file MCO2-5-e727-s001.docx]

**Supplementary Information For**

**Nicotinamide mononucleotide (NMN) supplementation rescues mitochondrial and energy metabolism functions and ameliorates inflammatory states in the ovaries of aging mice**

**Jinghui Liang^1^, Feiling Huang^1^, Xueyu Hao^2^, Peng Zhang^2^* and Rong Chen^1^***

^1^ Department of Obstetrics and Gynecology, Peking Union Medical College Hospital, Chinese Academy of Medical Sciences & Peking Union Medical College, National Clinical Research Center for Obstetric & Gynecologic Diseases, Beijing, China

^2^ Beijing Key Laboratory for Genetics of Birth Defects, Beijing Pediatric Research Institute; MOE Key Laboratory of Major Diseases in Children; Rare Disease Center, Beijing Children's Hospital, Capital Medical University, National Center for Children's Health, Beijing, China

*To whom correspondence should be addressed. Email: chenrongpumch@163.com (R.C.), zhangpengdyx@163.com (P. Z.).

**Supplementary Information-Figures：**

**
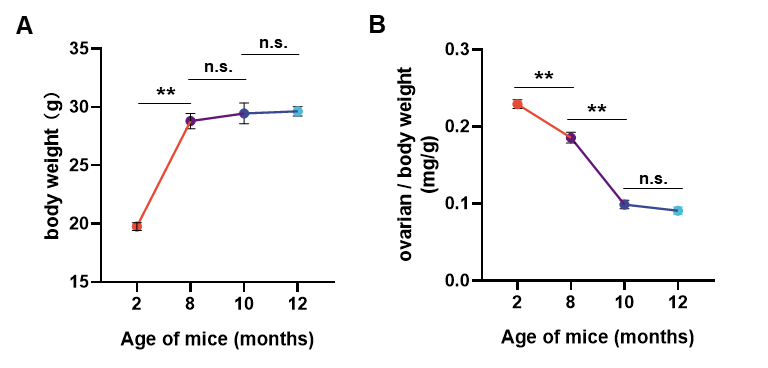
**

**Figure S1 Changes in mouse body weight and in the ovarian/body weight ratio during development.**

(A) Graph showing the body weights of the mice at different ages (n=17 mice at 2 months, 9 mice at 8 months, 6 mice at 10 months, and 14 mice at 12 months).

(B) Statistical analysis of ovarian/body weight ratios in mice of different ages (n=19 mice at 2 months, 13 mice at 8 months, 5 mice at 10 months, and 8 mice at 12 months).

The data are presented as the means ± SEMs. **, P < 0.01.


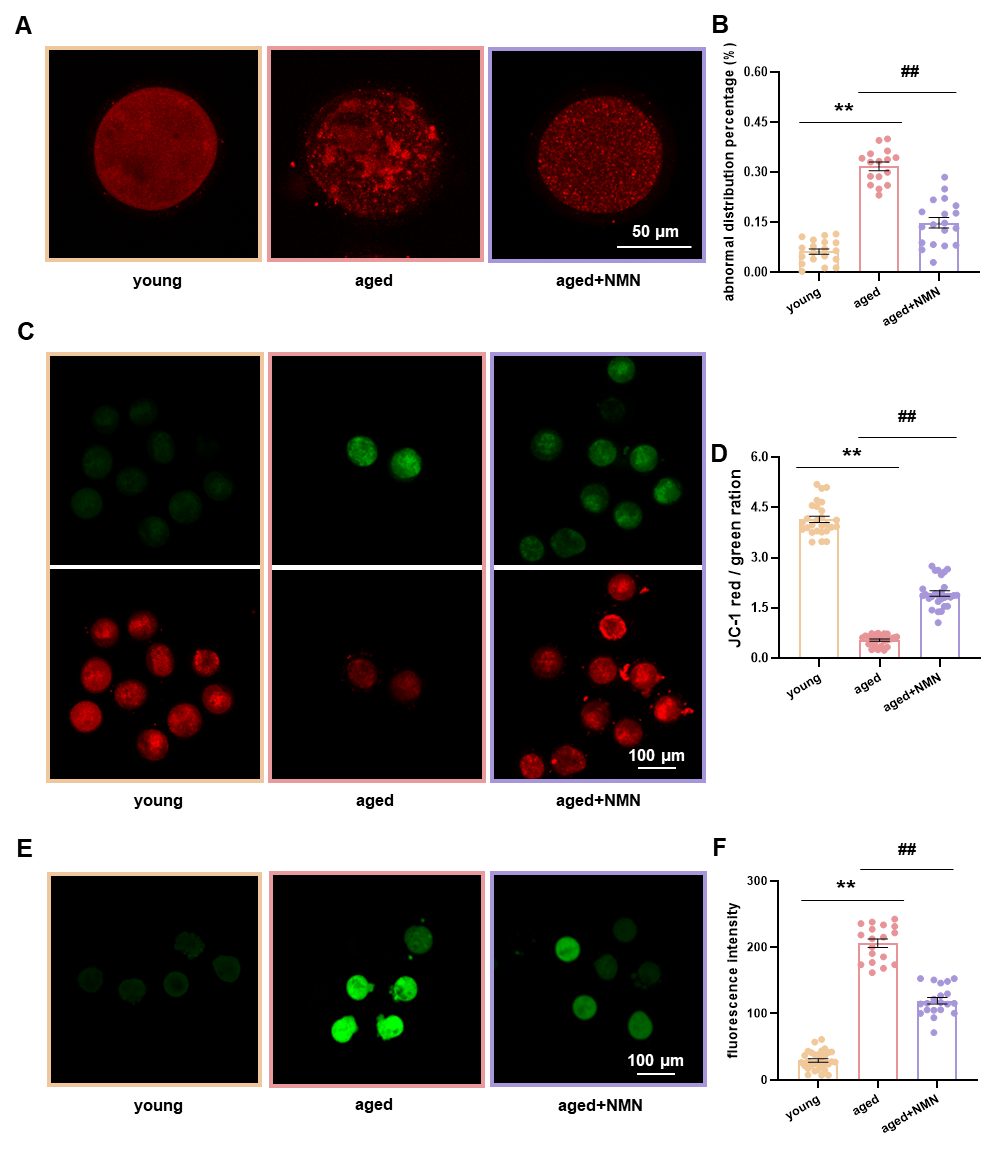


**Figure S2 Effects of NMN treatment on the quality of ovulation-induced oocytes from aged mice.**

(A) Representative images showing the distribution of mitochondria in oocytes from mice in the young, aged, and aged+NMN groups. The mitochondria in the oocytes were stained with MitoTracker Red. Scale bar, 20 μm.

(B) Statistical analysis of the proportion of oocytes from mice in the young, aged, and aged+NMN groups showing an abnormal distribution of mitochondria (n=19 follicles in the young and aged+NMN groups, 16 follicles in the aged group; ≥ 3 mice from each group).

(C) Representative images showing JC-1 staining in oocytes from young, aged, and aged+NMN mice. Red fluorescence indicates high mitochondrial membrane potential, and a green signal indicates low mitochondrial membrane potential. Scale bar, 200 μm.

(D) Mitochondrial membrane potential in oocytes from mice in the young, aged, and aged+NMN groups as determined by JC-1 staining (n=27 follicles in the young group, 23 follicles in the aged group, and 28 follicles in the aged+NMN group; ≥ 3 mice from each group).

(E) Representative images showing ROS levels in oocytes from mice in the young, aged, and aged+NMN groups visualized using an ROS detection kit. Scale bar, 200 μm.

(F) Statistical analysis of ROS levels in oocytes from mice in the young, aged, and aged+NMN groups (n=31 follicles in the young group, 18 follicles in the aged group, and 20 follicles in the aged+NMN group; ≥ 3 mice from each group).

The data are presented as the means ± SEMs. ** or ##, P < 0.01.


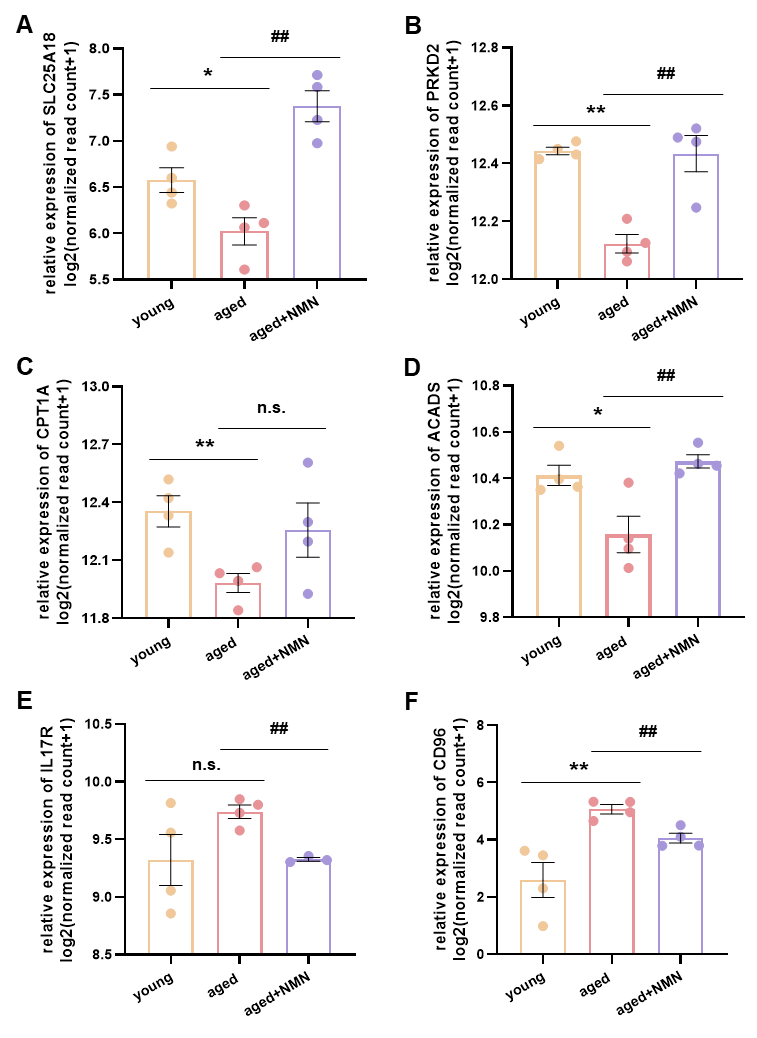


**Figure S3 DEGs in the ovarian transcriptome associated with mitochondrial function, energy metabolism, and immune pathways.**

(A-B) Changes in the expression of the mitochondrial function-related genes SLC25A18 and PRDX2 in the ovaries of mice in the young, aged, and aged+NMN groups (based on transcriptome data with read count+1 statistical results; n=4 mice per group).

(C-D) Changes in the expression of the fatty acid oxidation-related genes CPT1A and ACADS in the ovaries of mice in the young, aged, and aged+NMN groups (based on transcriptome data with read count+1 statistical results; n=4 mice per group).

(E-F) Changes in the expression of the inflammation-related genes IL17RA and CD96 in the ovaries of mice in the young, aged, and aged+NMN groups (based on transcriptome data with read count+1 statistical results; n=4 mice per group).
